# Resilience and spiritual well-being as resources for coping with radiotherapy and surviving in patients with glioblastoma

**DOI:** 10.1017/S1478951524001111

**Published:** 2024-11-05

**Authors:** Loredana Dinapoli, Morena Caliandro, Silvia Chiesa, Elisa Marconi, Nikola Dino Capocchiano, Ciro Mazzarella, Francesco Beghella Bartoli, Serena Bracci, Mario Balducci, Daniela Pia Rosaria Chieffo, Alba Fiorentino, Vincenzo Valentini, Luca Tagliaferri, Maria Antonietta Gambacorta, Nicola Dinapoli

**Affiliations:** 1 UOS di Psicologia Clinica, Fondazione Policlinico Universitario A. Gemelli IRCCS, Rome, Italy; 2 Dipartimento di Radioterapia Oncologica, Ente Ecclesiastico Ospedale Generale Regionale F. Miulli, Acquaviva delle Fonti (BA), Italy; 3 UOC di Radioterapia Oncologica, Dipartimento Diagnostica per Immagini, Radioterapia Oncologica ed Ematologia, Fondazione Policlinico Universitario A. Gemelli IRCCS, Rome, Italy; 4 Istituto di Scienze della Vita e di Sanità Pubblica, Università Cattolica del Sacro Cuore, Rome, Italy; 5 Dipartimento di Medicina, Università LUM, Casamassima (BA), Italy; 6 Centro di Eccellenza Oncologia Radioterapica, Medica e Diagnostica per Immagini, Ospedale Isola Tiberina-Gemelli Isola, Rome, Italy; 7 Università Cattolica del Sacro Cuore, Istituto di Radiologia, Rome, Italy

**Keywords:** Spiritual well-being, resilience, psychosocial distress, glioblastoma, radiochemotherapy

## Abstract

**Objectives:**

The primary aims of this multicenter, prospective observational study were to investigate spiritual well-being, resilience, and psychosocial distress in an Italian sample of glioblastoma patients undergoing radiochemotherapy. The secondary aim was to explore the influence of demographic, clinical, and psychological characteristics on survival.

**Methods:**

The assessment was conducted only once, within the first week of radiochemotherapy treatment. Spiritual well-being was evaluated by the Functional Assessment of Chronic Illness Therapy-Spiritual Well-being (FACIT-Sp-12), and religious/spiritual beliefs and practices were evaluated by the System of Belief Inventory. Resilience was evaluated by the Connor−Davidson Resilience Scale (CD-RISC). Psychosocial distress was evaluated the by Distress Thermometer and Hospital Anxiety Depression Scale. We conducted an univariable analysis of overall survival (OS) using data from the most recent follow-up available, considering demographic and clinical variables that could influence survival. Follow-up was defined as either the time of death or the latest follow-up visit recorded.

**Results:**

We recruited 104 patients, and the median follow-up time was 18.3 months. “Distressed” patients had lower scores than “not distressed” patients on the FACIT-Sp-12 and CD-RISC. While OS was not significant according to the FACIT-Sp-12 threshold, the Kaplan−Meier log-rank test was 0.05 according to the CD-RISC threshold. Among demographic variables, age showed significant associations with OS (*p* = 0.011). Resilience showed significant associations with OS (*p* = 0.025).

**Significance of results:**

Data showed that high spiritual well-being was associated with high resilience and an absence of psychosocial distress in our sample of glioblastoma patients undergoing radiochemotherapy. Patients with greater resilience survived longer than those with lesser resilience. Profiling spiritual well-being and resilience in glioblastoma patients undergoing radiochemotherapy can be seen as a resource to identify novel characteristics to improve clinical take-in-charge of glioblastoma patients.

## Introduction

Brain tumors are rare (3% of all tumors), but they are responsible for the highest loss of life years among all tumors (NICE). The most frequent malignant brain tumor is glioblastoma, with an estimated incidence rate of 3–4 cases per 100,000 inhabitants per year in Italy (AIOM). The average age of onset of glioblastoma is approximately 65 years (NICE). Even with new therapeutic advances and longer survival rates in patients with primary brain tumors, when compared with other cancers, the prognosis for glioblastoma is poor, with a median overall survival (OS) of 14.6–20.8 months with standard of care treatment and a survival rate of less than 5% at 5 years after diagnosis (Randazzo et al. 2021; AIOM).

Patients affected by glioblastoma are at a high risk of psychosocial distress due to the cognitive, behavioral, emotional, and functional deficits related to their disease (Trad et al. 2015). These patients show significant levels of anxiety and depression, which can also influence the survival outcome (Trad et al. 2015). Glioblastoma patients experience several radical challenges in their lives related to the uncertainty of their future and physical, psychological, and social changes, so most of them try to find relief through spirituality. The National Comprehensive Cancer Network defines spirituality as “a relationship between a person and a force or power beyond themselves that helps them feel connected and enriches their lives” (NCCN a). Spirituality can be nurtured through the practice of religious beliefs, prayer, and meditation and can be described as “a person’s sense of peace, purpose, connection to others, and beliefs about the meaning of life” (NCCN a). Literature data have demonstrated that spiritual well-being can help cancer patients struggling with physical and psychological symptoms by improving their coping strategies, strengthening their familial and friendship connections, and alleviating their distress (Randazzo et al. 2021). A large meta-analysis has shown positive associations between spiritual well-being and measures of physical, emotional, and social health (Sprik et al. 2021). The spirituality of cancer patients has been studied, especially among those in the terminal stage of disease or receiving palliative radiotherapy (Hematti et al. 2015; Piderman et al. 2014; Samuelson et al. 2012; Walshe et al. 2017). In the literature, only recently have studies been published on the standardization of specific tests to explore spiritual well-being in radiotherapy settings and to implement programs for spiritual well-being during radiotherapy (Elias et al. 2015; Henderson et al. 2012; Kouloulias et al. 2017; Miranda et al. 2020). However, research on spirituality in glioblastoma patients is lacking.

Psychological resilience is the dynamic process by which individuals harness their psychological, personality traits, and resources, along with biological and environmental factors, to protect and sustain their mental health amidst life’s adversities (Altinok et al. 2021; Fradelos et al. 2018; Tan et al. 2019). It involves positive adaptation within the context of significant challenges, reflecting an intricate interplay among various factors that mutually influence each other (Altinok et al. 2021; Fradelos et al. 2018; Tan et al. 2019). High resilience has been linked to positive health outcomes in cancer patients, such as lower rates of depression (Min et al. 2013; Sharpley et al. 2014). On the other hand, low resilience has been associated with poor psychopathological outcomes in cancer patients, such as increased rates of depression, anxiety, and psychological distress (Fradelos et al. 2017; Rosenberg et al. 2015). Resilience is considered a multifaceted process that encompasses a natural interaction of attributes, one of which is spirituality (Tan et al. 2019).

In our Radiotherapy departments, we routinely perform psychological assessments for glioblastoma patients. The mission is to help patients face the disease, cope with radiotherapy and its side effects and improve their quality of life (QoL) (Caliandro et al. 2023; Dinapoli et al. 2021). Patients can also benefit from personalized psychological support during radiotherapy (Dinapoli et al. 2021). Currently, no spiritual intervention is provided in our hospital, except for religious support. However, in the literature, there are increasing data on the efficacy of spiritual interventions for cancer patients, even during radiotherapy. Group therapy (Piderman et al. 2014), intercessory prayers (Miranda et al. 2020), mindfulness-based stress reduction programs (Henderson et al. 2012), and programs based on guided imagery/relaxation (Elias et al. 2015) have shown good results in improving QoL, spiritual well-being, and spiritual coping. To date, no data on spiritual well-being and resilience in glioblastoma patients during radiotherapy have been documented.

The primary aims of this study were to describe spiritual well-being, resilience, and psychosocial distress in glioblastoma patients undergoing radiochemotherapy and to evaluate whether spiritual well-being and resilience are differentially related to psychological and clinical characteristics, or cross-correlated.

The secondary aim was to investigate the impact of spiritual well-being and resilience on survival, considering specific interfering clinical variables (age, performance status, etc.).

## Materials and methods

This was an observational multicenter study. Two Italian centers were involved in the study: Fondazione Policlinico Universitario A. Gemelli IRCCS as a Coordinating center, and Ente Ecclesiastico Ospedale Generale Regionale Miulli, as a Participant center.

All consecutive patients with histologically proven isocitrate dehydrogenase 1 wild-type (IDH-1 wt) glioblastoma grading 4, with either complete or incomplete resection, those undergoing external beam radiotherapy with concurrent chemotherapy (radiochemotherapy) and those treated either by standard or hypofractionated regimens were enrolled in this study. Such criteria were adopted according to the recently updated publication of the World Health Organization (WHO) classification for brain tumors (Louis et al. 2021) to select appropriate glioblastoma patients. Patients who were able to understand and sign the informed consent form were considered eligible for this study. The exclusion criteria were the inability or denial to express informed consent, previously diagnosed major psychiatric disorders, and severe language deficits. Patients were treated according to the current treatment standards of the EORTC-NCIC regimen (Stupp et al. 2005) and the International Guidelines (NCCN b). Image acquisition, treatment planning, and radiotherapy were performed according to routinely used glioblastoma protocols in the Radiotherapy departments. All patients underwent either 3D conformal radiotherapy or volumetric modulated arc therapy (VMAT) with concurrent daily chemotherapy with temozolomide (TMZ) (daily TMZ at 75 mg/mq, from the first to the last day of radiotherapy). All patients received radiochemotherapy for the first time after their initial diagnosis. The Response Assessment in Neuro-Oncology criteria were adopted for analyses (Wen et al. 2010). Duration of follow-up was defined as the difference between the date of death and the date of diagnosis or (for patients alive at the time of analysis) the difference between the date of last visit and the date of diagnosis. Median follow-up was calculated using the Kaplan−Meier (KM) method.

### Psychological assessment

A thorough clinical psychological interview was conducted only once during the initial week of radiochemotherapy by psycho-oncologists from both Radiotherapy departments. During this session, the psycho-oncologists would infer whether or not the patient was able to understand the objective of the study and complete the questionnaires on their own.

Using a semi-structured clinical interview, the psychologists investigated the impact of diagnosis by exploring patients’ awareness of their illness, including their knowledge of histological data and the purpose of treatment. Then general areas of patient’s life were examined: personal history, psychopathological history, and lifestyle habits.

The spiritual sphere was investigated in the following manner: “Do you cultivate any spiritual sphere in your life? If so, how?” (For example: Catholic faith or other beliefs; believing in other higher powers). On this occasion, an assessment with specific tests was performed to evaluate psychosocial distress, spiritual well-being, and resilience. Total assessment time: 45 minutes.

The psychosocial distress (Wang et al. 2011) was assessed by the Distress Thermometer (DT) (Grassi et al. 2013) to evaluate emotional distress and the Hospital Anxiety and Depression Scale (HADS) (Zigmond and Snaith 1983) to evaluate mood. The DT is a visual analogue tool that is used to rate personal distress during the past week on a scale from 0 (no distress) to 10 (extreme distress). A DT cutoff score ≥4 is used to identify patients with emotional distress (“distressed patients”) (Grassi et al. 2013). The HADS (Zigmond and Snaith 1983) is a well-validated and reliable self-report measure designed to identify the presence and severity of anxiety and depression in cancer patients (Zigmond and Snaith 1983). It represents an effective screening tool for cancer patients undergoing treatment (Moorey et al. 1991). The HADS is a brief 14-item scale to report symptoms experienced by patients during the previous week. The HADS is divided into anxiety (HADS-A) and depression (HADS-D) subscales. Higher scores on either of the 2 subscales (depression and/or anxiety) indicate more severe symptoms. A global score ≥16 indicates anxiety/depression (“anxious/depressed patients”) (Moorey et al. 1991).

Spiritual well-being was evaluated by the Functional Assessment of Chronic Illness Therapy-Spiritual Well-being (FACIT-Sp-12) (Costantini et al. 2016). The FACIT-Sp-12 is a 12-item scale widely used in cancer patients (Costantini et al. 2016). The scale was developed to measure important aspects of spirituality, such as a sense of meaning in one’s life, harmony, peacefulness, and a sense of strength and comfort from one’s faith. It is divided into 3 dimensions, faith, meaning, and peace, distributed between 2 subscales (faith: 4 items; meaning/peace: 8 items). The score ranges from 0 to 4. The total score is the sum of the subscale scores, which ranges from 0 to 48, with a higher score indicating greater spiritual well-being (Costantini et al. 2016).

The System of Beliefs Inventory (SBI-15R) (Ripamonti et al. 2010) was used to collect useful information on the spiritual needs and resources of patients at any stage of the disease. The SBI-15R (Ripamonti et al. 2010), designed by Holland et al. (1998), measures religious and spiritual beliefs and practices and the social support derived from a community sharing those beliefs. The SBI-15R is a tool designed to elicit main religious beliefs (beliefs on the transcendence and transcendent meaning of human life) as well as attendance to religious practices and support received by the religious community (Holland et al. 1998; Ripamonti et al. 2010). The score of each item is determined by a 4-point Likert scale (from 0 to 3). Higher scores indicate higher levels of religiosity (Holland et al. 1998; Ripamonti et al. 2010).

Resilience was evaluated by means of the Connor−Davidson Resilience Scale (CD-RISC) (Connor and Davidson 2003; Tan et al. 2019). The CD-RISC is one of the most commonly used scales to measure resilience in cancer patients, consisting of 25 items that broadly map into the following 6 factors: (1) personal strength, (2) adaptability/flexibility, (3) self-determination, (4) giving best effort, (5) spirituality, and (6) social support (Connor and Davidson 2003; Tan et al. 2019).

This study was performed in line with the principles of the Declaration of Helsinki. Research Protocol (ID: 3420; Protocol Number 0035698) was approved by the Ethical Committee of our institution, Fondazione Policlinico Universitario A. Gemelli IRCCS.

### Sample size and statistical analysis

To explore the primary aim of the study, considering the historical case series of the 2 involved centers and the monthly access of glioblastoma patients to psychological services, a sample of at least 100 patients was predicted. With this lower bound of the predicted recruitable sample size, the collected data should be considered representative of the overall population (estimated for this calculation to be above 20,000 individuals) (AIOM) with a margin of error of 10% and a confidence level of 95%. To conservatively estimate the response distribution, a value of 50% was chosen. The sample size was calculated using the following formula:

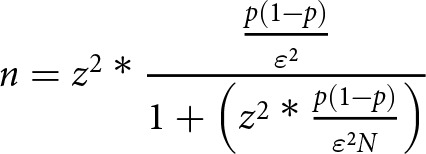



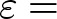
 margin of error; *N* = population size; *z* = *z* score of 1.96 for a confidence level of 95%; *p* = response distribution.

The clinical and demographic characteristics of the sample were described through an initial exploratory analysis and successive descriptive statistics. All statistical analyses were performed using the R statistical platform, and *p* values ≤ 0.05 were considered significant for statistical tests.

To investigate the primary endpoints, the following analyses were performed: the differences at CD-RISC, FACIT-Sp-12 and SBI-15 between “distressed” and “not distressed” and patients who exceeded/who did not exceed clinically significant cutoffs for anxiety/depression have been compared by Mann−Whitney univariable non parametrical test. The clinical and demographic variables were analyzed with respect to the psychological questionnaires with univariable statistical tests (Mann−Whitney for numeric or chi-square for categorical variables). Considering the possible cross correlation among the results obtained by all psychological tests and specific subscales of CD-RISC and FACIT-Sp-12, these have been analyzed using Pearson’s cross correlation test.

To investigate the secondary endpoint, an univariable survival analysis was performed by the KM test for factorial variables. For numeric variables, the best threshold was computed by calculating the lowest *p*-value using the KM test, moving the cutoff to split the population into 2 subpopulations within the range of observed values. The clinical variables interfering with survival in glioblastoma, according to the known predictive factors in the literature (age > 70 years (Gately et al. 2016), gender, Eastern Cooperative Oncology Group (ECOG), *O*(6)-methylguanine-DNA-methyltransferase (MGMT) methylation status, or surgery type (Stupp et al. 2014)), were investigated in the analysis.

Multivariable analysis was performed by the Cox proportional hazards (CPH) model after a cross-correlation test to determine unrelated variables.

## Results

### Overall demographic information

We recruited 104 patients (68 male and 36 female), with a median age of 59 years (see Table 1). The median follow-up time was 18.3 months. The whole sample was histologically homogeneous according to the latest WHO classification of brain tumors (Louis et al. 2021). All patients were able to sign the informed consent and easily fill the questionnaires.

**Table 1.** Clinical and demographic characteristics

Variable	*N* (%)
**Gender**	
Male	68 (65.4)
Female	36 (34.6)
**Age**	
Median/Mean/SD	59/58.8/±9.7
age	33−79
**Marital status**	
Married	88 (84.6)
Single	8 (7.7)
Widow/Widower	5 (4.8)
Divorced	3 (2.9)
**Working position**	
Retired	27 (26)
On sick leave	27 (26)
Currently working	19 (18.3)
Retired for the disease	17 (16.3)
Unemployed	8 (7.7)
Homemaker	6 (5.7)
**Histology**	
Glioblastoma multiforme	104 (100)
**MGMT methylation**	
Yes	34 (30.8)
No	62 (59.6)
NA	8 (9.6)
**Surgery type**	
Gross total resection	49 (47.1)
Partial resection/Biopsy	55 (52.9)
**Tumor site**	
Frontal right	11 (10.6)
Temporal right	19 (18.3)
Parietal right	5 (4.8)
Occipital right	5 (4.8)
Frontal left	10 (9.6)
Temporal left	15 (14.4)
Parietal left	10 (9.6)
Occipital left	3 (2.9)
Multicentric	3 (2.9)
Frontotemporal right	2 (1.9)
Frontotemporal left	1 (1)
Frontoparietal right	1 (1)
Frontoparietal left	3 (2.9)
Temporo-occipital left	2 (1.9)
Temporo-parietal right	5 (4.8)
Temporo-parietal left	5 (4.8)
Parieto-occipital left	2 (1.9)
Parieto-occipital right	2 (1.9)
**ECOG**	
0	51 (49)
1	45 (43.3)
2	7 (6.7)
3	1 (1)
**Steroids**	
Yes	72 (69.2)
No	32 (30.8)
**Antiepileptic drugs**	
Yes	96 (92.3)
No	8 (7.7)
**Believers in God**	
Yes	85 (81.7)
No	19 (18.3)
**Awareness of disease**	
Total	82 (78.8)
Partial	22 (21.2)
**Previous self-reported psychopathologies**	
Yes	20 (19.2)
No	84 (80.8)

MGMT *= O*(6)-Methylguanine-DNA-methyltransferase.

### Primary results

From the psychological interview, the following data emerged: the majority of patients, when asked “Do you cultivate a spiritual sphere?” responded affirmatively, stating that they believe in God (85 “believers” – 81.7%). No other credo was disclosed. Furthermore, the majority of patients were fully aware of the histological grade and the purpose of the radiochemotherapy treatment (82 out of 104 total awareness) (see Table 1).

Our cohort consisted of 34 (32.7%) “not distressed” patients and 70 (67.3%) “distressed” patients. The median overall DT score was 5 (range, 0–10). With respect to the HADS score, we identified 68 patients (65.4%) who did not exceed clinically significant cutoffs for anxiety/depression and 36 (34.6%) who exceeded clinically significant cutoffs for anxiety/depression. The median overall HADS score was 13 (range, 1–40).

“Distressed” patients had lower scores than “not distressed” patients on the CD-RISC and on both subscales of the FACIT-Sp-12 (see Table 2). Patients who exceeded clinically significant cutoffs for anxiety/depression had lower scores than those who did not on the CD-RISC and on both subscales of the FACIT-Sp-12 (*p* = 0.003; see Table 2).

**Table 2.** Wilkoxon signed-rank test’s results on distribution of CD-RISC, FACIT-Sp-12 over “distressed” patients and patients with HADS ≥16

		CD-RISC		FACIT meaning/peace		FACIT faith	
		Median (SD)	*p*	Median (SD)	*p*	Median (SD)	*p*
**DT**	Distressed (≥4)	67.0 (13.2)	**0.003**	22.0 (5.3)	**<0.001**	7.0 (5.0)	**0.001**
	Not distressed (<4)	79.5 (13.7)		27.0 (4.3)		12.0 (5.4)	
**HADS**	≥16	65.0 (13.0)	**<0.001**	21.0 (5.8)	**0.003**	5.5 (5.2)	**0.011**
	<16	76.5 (13.0)		24.0 (4.8)		9.0 (5.3)	

The results of the comparison among psychological tests and clinical/population features are summarized in Table 3. Of course, patients classified as “believers” showed a strong correlation with the FACIT-Sp-12 faith subscale and SBI-15R score, while married patients were found to be more resilient than unmarried patients (see Table 3).

**Table 3.** Univariable statistics among psychological tests and clinical/population variables

Variable	DT	HADS	FACIT-Sp-12 meaning/peace	FACIT-Sp-12 faith	CD-RISC	SBI-15R
Age^a^	0.580	0.878	0.995	0.690	0.524	0.258
Gender (male/female)	0.122	0.166	0.211	0.597	0.202	0.129
Marital status^b^	0.485	0.200	0.738	0.860	**0.035**	0.773
Working position^c^	0.611	0.435	0.060	0.246	0.420	0.391
Surgery type (gross total resection/partial resection)	0.819	0.353	0.720	0.275	0.351	0.160
ECOG^d^	0.216	0.202	0.510	0.385	0.930	0.492
Steroids (yes/no)	1.000	1.000	1.000	1.000	1.000	1.000
Antiepileptic drugs (yes/no)	0.347	0.328	0.082	0.416	0.302	0.705
Awareness of disease (total/partial)	0.421	0.437	0.252	0.533	0.265	0.699
Believers in God (yes/no)	0.956	0.784	1.000	**<0.001**	0.235	**<0.001**
Previous psychopathologies (yes/no)	0.052	0.390	0.337	0.303	0.152	0.723
Radiotherapy intent (radical/prolonged local control)	0.567	0.963	0.303	0.336	0.534	0.264

All binary variables have been used to split the scores of psychological tests into 2 populations, compared by Mann−Whitney test whose *p*-values are represented. The age^a^ variable has been split into 2 groups according to the median value (59 years); the marital status^b^ has been analyzed comparing *married* versus all the other conditions, because of the low number of cases in the other options in the list; the working position^c^ has been analyzed comparing patients *currently working* coupled with *on sick leave*, versus all the other options in the list; being ECOG^d^ 0 patients 51 in the entire population (49%), ECOG 1 were 45 (43.3%) and ECOG 2 and 3 only 8 (7.7%), we have compared the ECOG 0 population versus all the other conditions. These grouping among factors in variables ^b−d^ was needed because chi-square analysis was not feasible due to the small cases number in some of the options.

Temporal glioblastoma multiforme patients didnot show any difference at FACIT-Sp-12 (median total FACIT-Sp-12 score in temporal glioblastoma multiforme = 32 ± 9.3; median total FACIT-Sp-12 score in nontemporal glioblastoma multiforme = 31 ± 8.9; *p* = 0.77), thus excluding hyper-religiosity or religious delusions.

Pearson’s correlation test showed that the CD-RISC and FACIT-Sp-12 scores were strongly correlated (see Table 4). A correlation test was also performed between the SBI-15R score and the scores for all subdimensions of the FACIT-Sp-12 and CD-RISC (see Table 4).

**Table 4.** Matrix of Pearson’s cross correlation test results of SBI-15R, tests’ subscales of FACIT-Sp-12 and CD-RISC

			FACIT-Sp-12		CD-RISC					
		SBI-15R	Meaning/Peace	Faith	Personal strength	Spirituality	Social support	Adaptability/Flexibility	Giving best efforts	Self-determination
**SBI-15R**		–	**0.0385**	**<0.0001**	0.7891	**0.0001**	0.6549	0.1822	0.6392	0.4181
**FACIT-Sp-12**	**Meaning/Peace**	**0.0385**	–	**<0.0001**	**<0.0001**	0.2627	**0.0005**	**<0.0001**	**0.0207**	**<0.0001**
	**Faith**	**<0.0001**	**<0.0001**	–	0.2114	**<0.0001**	0.1893	**0.0025**	0.5020	**0.0082**
	**Personal strength**	0.7891	**<0.0001**	0.2114	–	0.0541	**0.0492**	**<0.0001**	**<0.0001**	**<0.0001**
	**Spirituality**	**0.0001**	0.2627	**<0.0001**	0.0541	–	0.3972	**<0.0001**	**0.0116**	**0.0003**
**CD-RISC**	**Social support**	0.6549	**0.0005**	0.1893	**0.0492**	0.3972	–	**0.0109**	0.4643	**0.0125**
	**Adaptability/Flexibility**	0.1822	**<0.0001**	**0.0025**	**<0.0001**	**<0.0001**	**0.0109**	–	**<0.0001**	**<0.0001**
	**Giving best efforts**	0.6392	**0.0207**	0.5020	**<0.0001**	**0.0116**	0.4643	**<0.0001**	–	**<0.0001**
	**Self-determination**	0.4181	**<0.0001**	**0.0082**	**<0.0001**	**0.0003**	**0.0125**	**<0.0001**	**<0.0001**	–

### Secondary results

**Figure 1.**
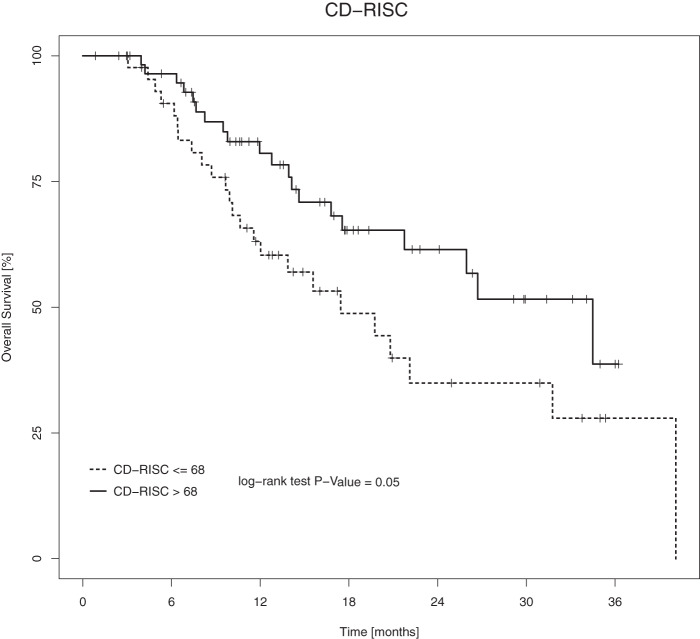
Survival curves of the patient population classified against the threshold of CD-RISC 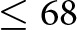 or higher. The log-rank test result is 0.05.

OS was determined by the difference between the time of death or the time of the last available follow-up (for surviving patients) and the time of histological diagnosis. Time of death was obtained from medical records or data available in the National Health System Registry. The OS analysis updated at the last available follow-up (*N* = 104) showed a median OS time of 22.1 months. This analysis revealed that patients with lower spiritual well-being and lower resilience had shorter survival times than patients with higher spiritual well-being and higher resilience. The best performing cutoffs were a FACIT-Sp-12 score > 32 and a CD-RISC score > 68. While OS according to the FACIT-Sp-12 threshold was not significant, the KM log-rank (LR) test was 0.05 according to the CD-RISC threshold (Fig. 1). SBI-15R scores were not significantly related to OS. Moreover, OS was significantly correlated with age (cutoff of 70 years, *p* = 0.04), while OS did not show a correlation with sex, MGMT methylation or surgery type in our cohort (see Table 5). An age > 70 years was not cross-related with either low resilience or low spiritual well-being (Fisher’s test *p* > 0.05). Resilience and spiritual well-being were found to be cross-related, so only the most significant variable (resilience) with a lower *p*-value in the KMLR test was included in the multivariate analysis. CPH showed that both age and resilience were significantly related to OS (*p* = 0.011; *p* = 0.025) (see Table 6).

**Table 5.** Descriptive statistics and univariable survival analysis of patients’ population (Kaplan−Meier)

Variable	*p*
Gender	0.090
Age	**0.040**
Marital status	0.300
Working position	0.100
Surgery type	0.200
MGMT methylation	0.900
ECOG	0.600
Steroids	0.200
Antiepileptic drugs	0.300
Believers in God	0.900
Awareness of disease	0.090
Previous self-reported psychopathologies	0.900

Only the age is significant considering the threshold of 70 years age as cutoff to split the population used previously in literature (Gately et al. 2016).

MGMT = *O*(6)-Methylguanine-DNA-methyltransferase.

**Table 6.** Results of multivariable analysis (Cox proportional hazards test)

	coef	exp (coef)	Lower .95	Upper .95	SE (coef)	*z*	*p*-value
Age	0.04729	1.04843	1.0110	1.0872	0.01853	2.552	**0.011**
CD-RISC > 68	−0.69267	0.50024	0.2726	0.9181	0.30979	−2.236	**0.025**
		Concordance = 0.656 (SE = 0.046)					
		Likelihood ratio test = 10.41 on 2 *df*					**0.005**
		Wald test = 0.14 on 2 *df*					**0.006**
		Score (log-rank) test = 10.05 on 2 *df*					**0.007**

Both age and CD-RISC > 68 return significant *p*-values.

## Discussion

After a cancer diagnosis, patients are prone to physical, psychological, social, and spiritual changes that may be related to anxiety, depression, and hopelessness (Cheng et al. 2019). Previous studies have confirmed that spirituality is an important coping resource for cancer patients to adjust to their disease (Jimenez-Fonseca et al. 2018) Spirituality can become “an inner resource or inner aspect of a person” that is efficacious for coping with main stressors (Chaar et al. 2018). Spiritual well-being and psychological resilience (Chaar et al. 2018) are known to play an important role in individuals’ attempts to cope with adverse events; in fact, breast cancer survivors with high spiritual well-being and psychological resilience showed less fear of recurrence during follow-up (Chaar et al. 2018). Despite growing data about the role of psychological characteristics in outcomes, such topics have not been addressed extensively in patients with brain tumors. Interventions in this field have shown that writing a spiritual legacy document induced favorable changes in serenity and positive religious coping (Piderman et al. 2017). Strang and Strang (2001) found that meaningfulness was central to brain tumors patients’ QoL and was created by close relations and faith. A recent study on a large cohort of patients with brain tumors ranging from WHO grade <III to stage IV (Randazzo et al. 2021) showed that spiritual well-being was an independent predictor of QoL within the brain tumor population. In our study, patients with a nonsignificant level of psychosocial distress showed higher resilience and spiritual well-being, thus suggesting a relationship between being resilient or spiritual and having low psychosocial distress. In general, spiritual well-being and resilience were correlated in our sample, similar to another recent study in advanced cancer patients (Mihic-Góngora et al. 2022).

Numerous studies support the relationship between meaning/peace and a variety of outcomes: meaning/peace is related to coping (Merluzzi et al. 2023), or, in a study of cancer survivors, low scores on the meaning/peace subscale, but not the faith subscale, were associated with depressive symptoms (Mihic-Góngora et al. 2022). Moreover, even in the context of high levels of faith, concomitant low levels of meaning resulted in greater depressive symptoms (Bamishigbin et al. 2020). In another study, higher baseline meaning/peace scores and an increase in these scores over time predicted a decrease in depressive symptoms, less distress, and greater vitality after 12 months in breast cancer patients compared to no increase in scores (Yanez et al. 2009). To our knowledge, our paper is the first to report that meaning/peace is related to almost all the components of the resilience scale. Thus, our glioblastoma patients are characterized by 2 strong attributes: (1) The ability to make sense of their lives by leaning on facets of strong psychological resilience and (2) faith, which had not relented at the time the patients were interviewed, with no patient declaring any struggle with God while in a proactive phase of their path; indeed, faith was a key resource in coping with such a long radiochemotherapy treatment period.

To date, no study has evaluated the role of spiritual well-being and resilience in glioblastoma patients undergoing radiochemotherapy and their possible impact on survival. Our data showed that patients with higher resilience showed benefits in terms of survival compared with patients with lower resilience. These results, if confirmed in more structured research studies, should reflect on the implementation of psychological, personalized monitoring and interventions for glioblastoma patients, with the objective of increasing such resources. The implications of our study are as follows: screening for psychological characteristics is important to identify glioblastoma patients who might be struggling and could benefit from psychosocial support, given that resilience seems related to medical outcome. In our previous study (Dinapoli et al. 2021), distressed glioblastoma patients had a reduced median survival time compared with “not distressed” glioblastoma patients. This observation was not confirmed in the present study, likely due to the characteristics of the actual sample size and study design. The association between spiritual well-being and psychosocial well-being found in other studies (Bai et al. 2015; Chaar et al. 2018: Randazzo et al. 2021) was confirmed by our data.

Our study investigated a topic that is underexplored in the literature, although scientific interest in glioblastoma patients is growing. The limitations of the present study are related to our study design. A comparison with other histologies or diseases may produce more significant data. Resilience is a dynamic process (Baksi et al. 2021) and may change depending on conditions. Additionally, comparisons among patients with different pathologies could provide various points of interest, especially for survival outcomes. Of course, the evaluation of spiritual well-being, resilience, and psychosocial distress in an early stage of disease would benefit from a longitudinal assessment. Certainly, one of the limitations of the study is that it has an observational design, with possible subsequent enrolment biases. Therefore, the absence of a correlation between MGMT methylation status, extent of surgery, and survival, such as in our case series, is not a general proof of their lack of correlation. This discrepancy could affect the validity and generalizability of the findings about survival, underscoring the need for cautious interpretation and further research about this topic. On the other hand, the main objective of our study was to evaluate the spiritual well-being and resilience of glioblastoma patients, and the study has shown us the association between these variables and patients’ psychosocial distress.

Psychological support in glioblastoma patients should integrate the empowerment of resilience to elicit spiritual resources and help patients better cope with such a dismal diagnosis. As psycho-oncologists on both topics, we should focus on developing an empathic presence, reflective listening, and the administration of self-care interventions (meditation, yoga, gratitude diary, life review) in the future. Cognitive behavioral therapy, mindfulness-based psychotherapy, and resilience training programs based on the positive psychology approach and on supportive–expressive group therapy can also enhance these constructs (Ludolph et al. 2019). Another approach could be taken from Eye Movement Desensitization and Reprocessing (EMDR) psychotherapy by using the Resource Development Installation technique (Korn and Leeds 2002) to elicit spiritual and resilience resources. Specific EMDR cancer protocol is useful to help patients resume personal control over aspects experienced as unmanageable (Faretta and Borsato 2016); in fact, in initial phase of the protocol the objectives are improving self-care, and coping skills and empowering resilience (Faretta and Borsato 2016). Moreover, recent data showed the beneficial effects of EMDR in alleviating affective symptoms and improving meaningfulness, comprehensibility, and manageability of glioblastoma patients’ lives (sense of coherence) (Szpringer et al. 2018). Additionally, the implementation of spiritual support should be encouraged to provide relief for patients of all faiths in our hospital and create spaces and referral figures for each spiritual denomination. We can hypothesize that spirituality and resilience are “instrumentally linked” in cancer patients, with spirituality being a continuously evolving process similar to a “pathway” to resilience (Manning 2013). Other factors such as social connection and internal resources, including a direct approach to challenges, curiosity, and extending oneself to others, should be explored and enhanced in order to complement spirituality in a perspective of increasing resilience (Kinsel 2005).

Investigating spirituality and resilience can also help health-care professionals implement an individualized intervention plan based on patients’ values, preferences, resources, and needs. Examining spiritual well-being and resilience among glioblastoma patients undergoing radiotherapy can yield valuable insights for identifying novel prognostic indicators and improving clinical pathways tailored to the specific needs of these patients. In the near future, knowledge about spiritual well-being and resilience in these patients could lead to (1) personalized psychological interventions during radiotherapy or according to the disease trajectory and (2) personalized spiritual or resilience interventions for glioblastoma patients.
